# Varied presentations of pancreatic insulinoma: a case report

**DOI:** 10.11604/pamj.2022.42.69.34839

**Published:** 2022-05-25

**Authors:** Pueya Rashid Nashidengo, Francis William Quayson, John Tabiri Abebrese, Lahja Negumbo, Cornell Enssle, Fredrick Kidaaga

**Affiliations:** 1 Department of Surgery, Windhoek Central Hospital, Windhoek, Namibia,; 2 Department of Radiology, Windhoek Central Hospital, Windhoek, Namibia,; 3 Namibia Institute of Pathology, Windhoek, Namibia

**Keywords:** Insulinoma, neuroendocrine tumours, tumour enucleation, COVID-19, case report

## Abstract

An insulinoma is a rare functional pancreatic neuroendocrine tumour that is usually sporadic and solitary. The hallmark is hypersecretion of insulin, which leads to neuroglycopenia symptoms and uncontrolled sympathoadrenal activity. Neuroendocrine tumours can have a varied presentation, with symptoms often ascribed to a different diagnosis, thus delaying correct diagnosis and treatment. We present the case of a 26-year-old female who had a 3-year delay before diagnosing insulinoma after being initially assessed with epilepsy and schizophrenia. The case report below provides a detailed review of the diagnosis, tumour localization, and surgical interventions implemented for the patient during the COVID-19 pandemic.

## Introduction

Pancreatic neuroendocrine tumours (NET) are rare lesions with a reported incidence of four cases per one million patients per year [1]. An international survey of 1928 patients with NETs reported a mean delay of 52 months between symptom onset and diagnosis. According to the study, patients see an average of six different health care providers before receiving the correct diagnosis [2]. Insulinomas are the most common functioning endocrine neoplasm of the pancreas. As clinical manifestations are non-specific, diagnosing an insulinoma is challenging and greatly depends on the findings from a thorough patient and collateral history and a high index of suspicion. Insulinoma is suggested by Whipple's triad, which is the presence of hypoglycemic symptoms, documented hypoglycemia and resolution of the hypoglycemic symptoms after glucose administration [3]. Biochemical confirmation of hyperinsulinism uses a supervised 72-hour fasting test with plasma glucose, insulin, C-peptide, and proinsulin level measurements. This method remains the gold standard of diagnosis [4].

Following confirmation of hyperinsulinism, the next step is tumour localization to aid surgical planning. Choice of imaging modality is dependent on the availability of resources and local radiologic expertise. The use of an open or laparoscopic approach depends on tumour size, location and resource setting [5]. Tumour enucleation is the procedure of choice, especially in a small and solitary nodule. Enucleation is sufficient in a well-circumscribed lesion that is preoperatively localized. Pancreatic resection is indicated for lesions invading or in close proximity to the pancreatic duct or major vessels, or suspicious for malignancy with a hard, infiltrating tumour and puckering of the surrounding soft tissue, pancreatic duct dilatation or lymph node involvement [3].

## Patient and observation

**Patient information:** a 26-year-old female, diagnosed with schizophrenia and epilepsy in her medical records, presented to a local clinic accompanied by neighbours with a history of loss of consciousness for an unknown duration. At presentation, her random blood glucose (RBG) was 1.1mmol/litre (normal range 3.7-6.1mmol/litre). The hypoglycemia was corrected with an intravenous bolus of 50% dextrose, followed by an infusion of 5% dextrose. The patient regained consciousness after the treatment but became unresponsive approximately 8 hours later with another episode of hypoglycemia which prompted a transfer to a tertiary referral hospital following correction of the hypoglycemia. At the emergency department of the referral hospital, she had visual and auditory hallucinations with inappropriate behaviour. Her blood glucose reading was 1.8mmol/L, and dextrose boluses and continuous infusion were administered with symptom resolution. She had a 3-year history of recurrent episodes of loss of consciousness, confusion, seizures, and bizarre behaviour. Symptom onset was during periods of fasting, often in the mornings, and resolved after food intake. Additionally, there was polyphagia with significant weight gain. Within this period, she was diagnosed with schizophrenia and epilepsy and treated with chlorpromazine, sodium valproate and phenobarbitone.

**Clinical findings:** the patient's vital signs were within normal limits and she had a BMI of 32.0 kg/m^2^ (height 175cm, weight 98kg). Systemic examination was otherwise unremarkable.

**Timeline:** from the patient's narration and collateral history, her symptoms, initially mild and consisting of generalized weakness and polyphagia, began four years prior to presentation. It had progressively worsened to include multiple episodes of loss of consciousness, mainly occurring in the morning, aggressive behaviour, seizures and amnesia. She was initially diagnosed with epilepsy and then schizophrenia at a local hospital and given treatment for both. However, there was minimal improvement in her symptoms thus prompting her referral to a tertiary hospital. A 24-hour fasting test with fasting blood glucose, insulin and C-peptide levels yielded a provisional diagnosis of insulinoma. An initial tumour localization with computed tomography (CT) was performed and followed up with endoscopic ultrasound and fine-needle aspiration cytology of the mass, which confirmed an insulinoma. The patient tested positive for COVID-19 but was clinically asymptomatic and was isolated for 10 days; as per national COVID-19 guidelines. Subsequently, enucleation of the pancreatic tumour was done with 24-hour postoperative monitoring in the intensive care unit (ICU). The patient was discharged home following an unremarkable postoperative period and a multidisciplinary team review with complete resolution of her pre-surgery symptoms.

**Figure 1:**
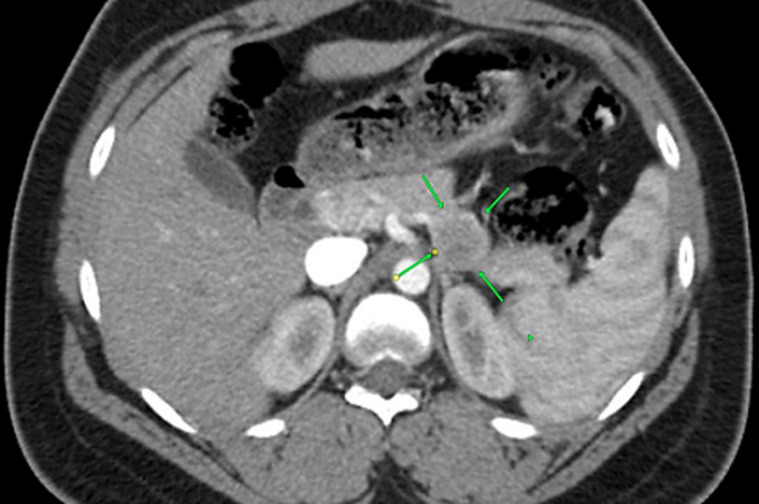
computed tomography, axial view of the tumour

**Figure 2:**
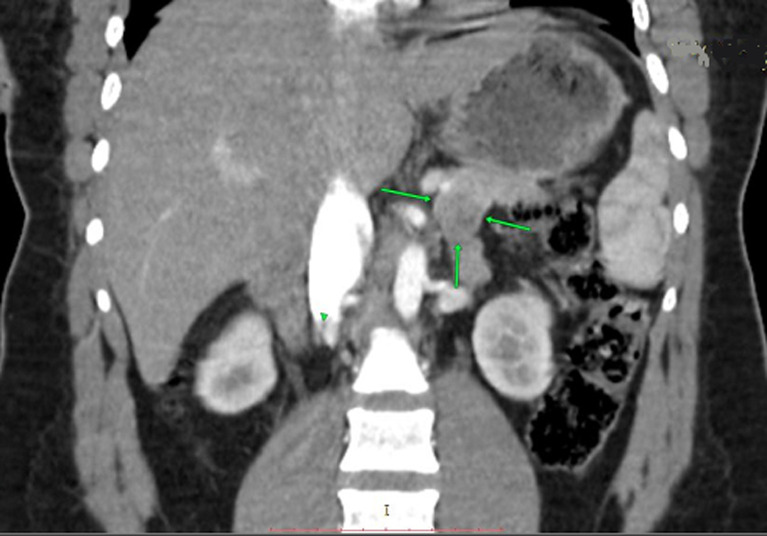
computed tomography, coronal view of the tumour

**Diagnostic assessment:** the patient met Whipple's criteria, and we used a 24-hour fast with blood glucose, insulin, and C-peptide levels to investigate the differential of an insulinoma. She had a blood glucose level of 18 mmol/l, an 81.1 µIU/mL insulin level, and a C-peptide of 2969 pmol/L after a 24-hour fast (Table 1). The results confirmed the diagnosis of insulinoma. For localization of the insulinoma, computed tomography was the initial imaging used, followed by endoscopic ultrasound (EUS) with a EUS-guided fine needle aspiration cytology (FNAC). The CT demonstrated a 2.5 cm by 2.4 cm well-circumscribed hypodense tumour in the body-tail region of the pancreas, with no lesions suggestive of lymph node involvement or metastases noted in other organs (Figure 1, Figure 2). The endoscopic ultrasound revealed a hyperechoic tumour with measurements and localization similar to the CT findings. In addition, the cytological exam of the FNA material from the tumour demonstrated cells with neuroendocrine features.

**Table 1:** laboratory results after 24-hour fast

	24-hour fast result	Normal fasting reference range
Glucose (mmol/l)	1.8	<3.9
Insulin (μU/mL)	81.1	<11.1
C peptide (pmol/l)	2969	<1270

**Therapeutic intervention:** the patient was scheduled for laparotomy and enucleation of the insulinoma. During the preoperative period a continuous glucose infusion was administered, to achieve symptom control, due to the unavailability of diazoxide. Laparotomy was performed with enucleation of the insulinoma after division of the gastrocolic ligament and inferior mobilization of the pancreas. The tumour was localized to the body-tail region of the pancreas, posteroinferior to the gland in keeping with prior imaging. The patient was admitted to the ICU for 24-hour monitoring and discharged to the general surgical ward. Following enucleation, the patient had complete resolution of her symptoms with an uneventful postoperative period. A multidisciplinary team which included: oncologists; general surgeons and radiologists reviewed the patient, and the consensus was that surgery was curative. The patient was discharged for follow-up care at the regional hospital and scheduled for an abdominal CT at six months post-surgery.

**Figure 3:**
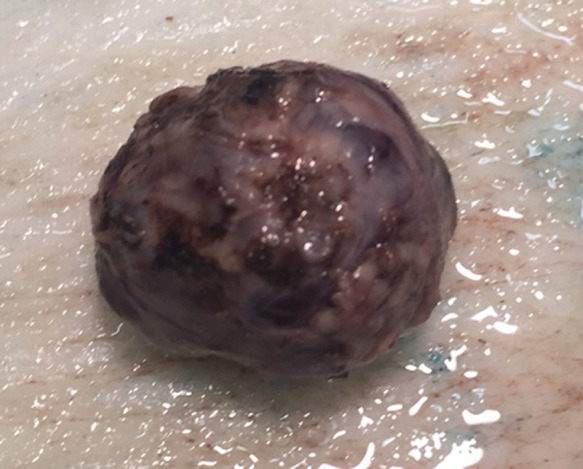
macroscopic image of the insulinoma (whole)

**Figure 4:**
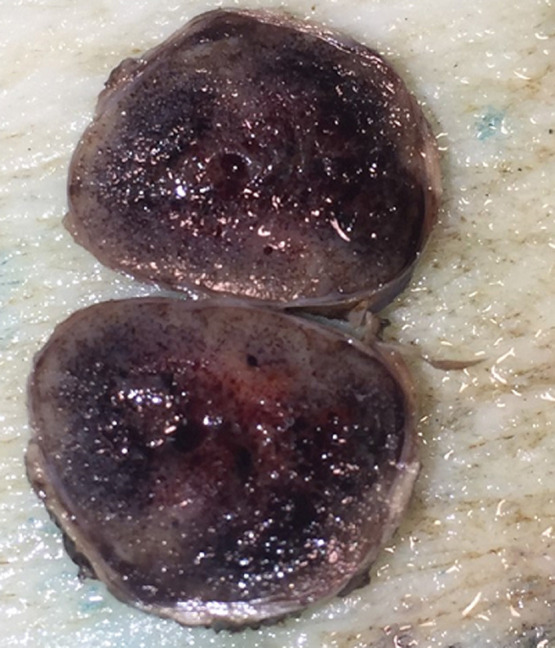
macroscopic image of the insulinoma (cut)

**Figure 5:**
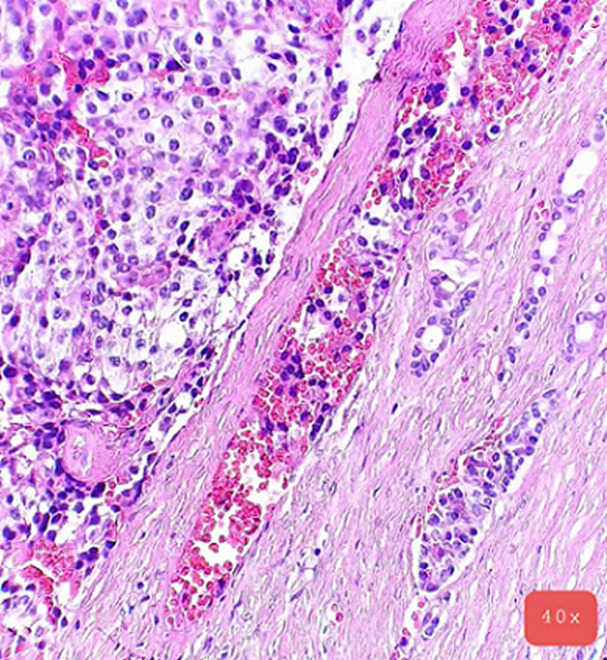
microscopic image with H&E stain (40x magnification)

**Figure 6:**
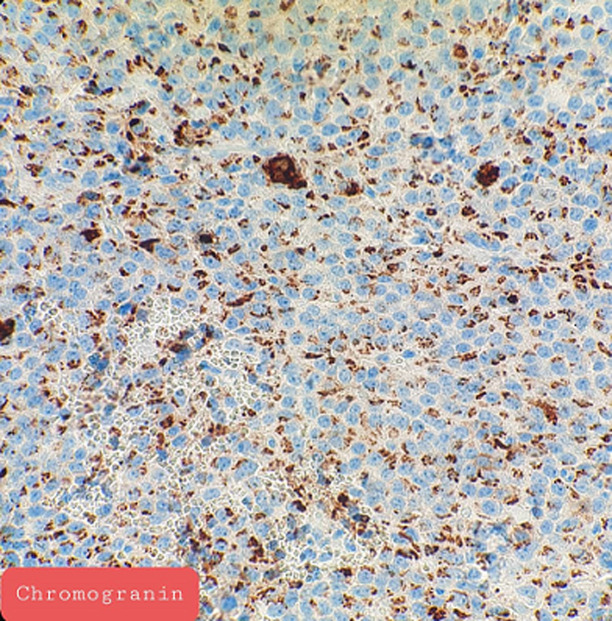
immunostaining of insulinoma

**Pathology report:** macroscopically, the tumour was nodular, well-circumscribed, encapsulated and measured 2.5cm in diameter. The cut surface was hemorrhagic (Figure 3, Figure 4). Microscopically, a monotonous population of medium-sized round to oval cells were observed. The tumour cells were arranged in solid nests and acini. The nuclei were centralized, had inconspicuous nucleoli and with salt and pepper chromatin. The cytoplasm was eosinophilic and moderately abundant. No atypia and mitoses were noted. The tumour was highly vascularized. Lymphovascular invasion was not evident. Immunohistochemically, the cells were positive for neuroendocrine markers (Figure 5, Figure 6). A well-differentiated neuroendocrine tumour was diagnosed histologically (WHO grade 1).

**Follow-up and outcome of interventions:** the patient had been weaned off epilepsy and schizophrenia medications without incident. In addition, she had started a diet and exercise-based regimen to achieve weight loss.

**Patient perspective:** based on patient and collateral reports, there has been a significant improvement in the patient's functionality since the surgery. For example, the patient no longer experiences easy fatiguability, periods of increased appetite, seizures, or loss of consciousness.

**Informed consent:** we obtained consent from the patient for the publication of this article.

## Discussion

Insulinomas are functional neuroendocrine tumours that develop from pancreatic beta islet cells. Insulinomas are described in the literature using the 90% characteristic rule, which states that 90% are benign, 90% are solitary, and 90% are small (less than 2cm in diameter) [4]. Up to 10% of insulinoma cases are linked to multiple endocrine neoplasia type 1 (MEN1), an autosomal dominant disorder due to a mutation in the MEN1 gene [1]. The clinical diagnosis is suspected following documentation of Whipple's triad, which is defined as the presence of hypoglycemic symptoms, biochemical demonstration of hypoglycemia (serum glucose <2.5 mmol/L), and remission of hypoglycemic symptoms following glucose administration [6]. Fasting serum glucose levels ( <2.5 mmol/L), insulin (36 pmol/L or more by radioimmunoassay), proinsulin (5 pmol/L or higher), and C-peptide levels (200 pmol/L or more) should all be measured. Increased C-peptide levels support evidence of endogenous insulin synthesis, which is consistent with the diagnosis of insulinoma. In the presence of exogenous insulin, C-peptide levels are typically decreased [7]. A 72-hour fast is required to induce and collect the biochemical data needed to demonstrate a hyperinsulinemia hypoglycemic condition. Insulinomas, regardless of their benign anatomical qualities, can be fatal. Neuroglycopenic symptoms include behavioural changes, visual disturbances, weakness, confusion, dizziness, seizures, and loss of consciousness. If not addressed, these can lead to coma and death. Palpitations, diaphoresis, tremors, and anxiety are examples of neurogenic symptoms [3].

Some patients present to the health care system with recurrent neurological or psychiatric symptoms, resulting in misdiagnoses with epilepsy or a psychiatric condition. These symptoms become typically evident after fasting and are often precipitated by physical exercises. However, the median duration of symptoms before diagnosis remains variable and can reach 12-18 months on average or even years in rare cases [8]. It took three years for our patient to be diagnosed with insulinoma after an initial diagnoses of epilepsy and schizophrenia for which she was treated with antiepileptic and antipsychotic medications with no improvement in symptoms. Pre-operative tumour localization ensures optimal surgical preparation. Localization methods can be both invasive and non-invasive. Laparoscopic ultrasounds can help with tumour localization intraoperatively [3]. Although not commonly used, magnetic resonance imaging can detect liver metastases [4].

Surgery is the definitive treatment for insulinomas, with cure rates ranging from 77 to 100%. Ninety percent of insulinomas are benign, solitary, and small (less than 2 cm in diameter) therefore pancreas-sparing techniques like enucleation and partial pancreatectomy are preferred. This can be done via an open or laparoscopic approach. The extent of surgery is dependent on the tumour's location and its malignant potential [5]. Non-surgical options exist for pre-operative control of serum glucose levels, patients not fit to tolerate anaesthesia or those with uncontrolled disease. Pharmacological management of insulinoma includes the use of diazoxide, calcium channel blockers, propranolol and glucocorticoids. Octreotide is a somatostatin analogue that inhibits insulin secretion and is helpful in the treatment of malignant or unresectable insulinomas [9]. Patients diagnosed with benign insulinomas have an excellent prognosis, all returning to normal life after treatment [10].

## Conclusion

Insulinomas are benign and curable in most cases but can be fatal if misdiagnosed. In addition, Insulinomas share common clinical manifestations with epilepsy and various psychiatric conditions; it is for this reason that insulinomas should be included in the differential diagnosis of epilepsy and other psychiatric diagnoses to prevent misdiagnosis.
